# Supplementary material for: Yeast MoClo Secretion and Surface Display Toolkit 2.0: Improvements and Applications for Analysis of protein–protein Interactions and Whole-Cell Biocatalysis
Source: ACS Synth Biol. 2026 Apr 23;15(5):2032–53. doi: 10.1021/acssynbio.6c00085 (PMC13185157; doi:10.1021/acssynbio.6c00085)
Supplement: Supplementary file 1 [file sb6c00085_si_001.pdf]

SUPPORTING INFORMATION

(SUPPORTING FIGURES AND TABLES)

Yeast MoClo secretion and surface display toolkit 2.0: improvements and applications for analysis of protein-protein interactions and whole-cell biocatalysis

AUTHORS

Vanja Jurić[1,2], Leah G. Erwin[1], Nicola M. O'Riordan[1], Eamonn Maher[1,2], Justin D. Holmes[2,3], Paul W. Young[1,2,*]

AFFILIATIONS

[1]School of Biochemistry and Cell Biology, University College Cork, Cork T12 YN60, Ireland

[2]AMBER Centre, Environmental Research Institute, University College Cork, Cork T23 XE10, Ireland

[3]School of Chemistry, University College Cork, Cork T12 YN60, Ireland

*Correspondence: p.young@ucc.ie

Figure S1

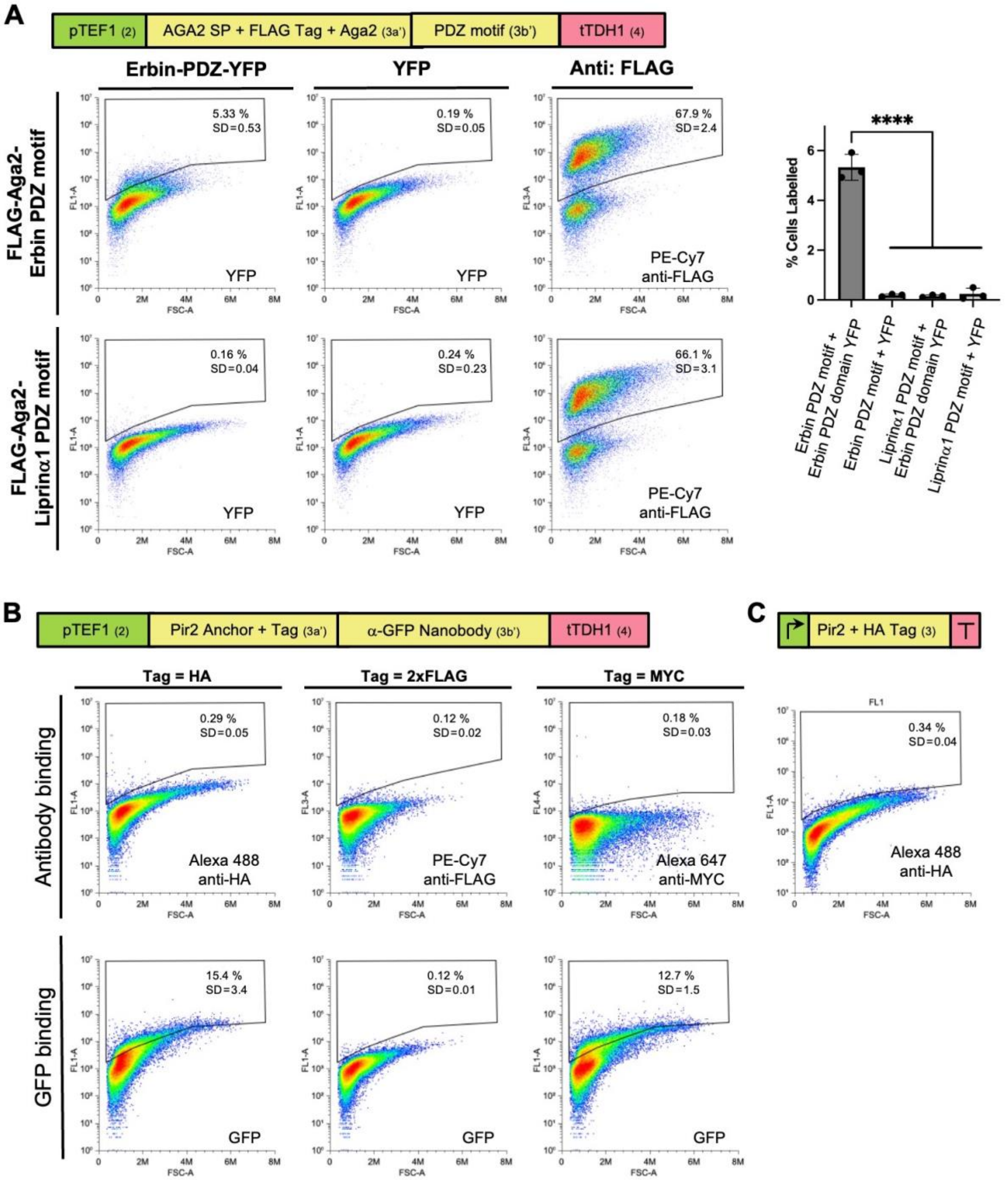
A
pTEF1 (2)
AGA2 SP + FLAG Tag + Aga2 (3a')
PDZ motif (3b')
tTDH1 (4)
Erbin-PDZ-YFP
YFP
Anti: FLAG
FLAG-Aga2-Erbin PDZ motif
FLAG-Aga2-Liprinα1 PDZ motif
5.33 % SD=0.53
0.19 % SD=0.05
67.9 % SD=2.4
0.16 % SD=0.04
0.24 % SD=0.23
66.1 % SD=3.1
PE-Cy7 anti-FLAG
% Cells Labelled
****
Erbin PDZ motif + Erbin PDZ domain YFP
Erbin PDZ motif + YFP
Liprinα1 PDZ motif + Erbin PDZ domain YFP
Liprinα1 PDZ motif + YFP
B
pTEF1 (2)
Pir2 Anchor + Tag (3a')
α-GFP Nanobody (3b')
tTDH1 (4)
Tag = HA
Tag = 2xFLAG
Tag = MYC
Antibody binding
GFP binding
0.29 % SD=0.05
0.12 % SD=0.02
0.18 % SD=0.03
Alexa 488 anti-HA
PE-Cy7 anti-FLAG
Alexa 647 anti-MYC
15.4 % SD=3.4
0.12 % SD=0.01
12.7 % SD=1.5
GFP
C
Pir2 + HA Tag (3)
0.34 % SD=0.04
Alexa 488 anti-HA
FSC-A

Figure S1 Characterization of new anchors added to the MoClo yeast surface display toolkit
(A) Detection of the interaction of a PDZ domain with a PDZ-binding motif displayed as a carboxyl terminal fusion to Aga2. Yeast strains were generated that displayed either a motif that is known to bind to the Erbin PDZ domain *(1)*, or an unrelated PDZ-binding motif from Liprin-α1 *(2)*, as carboxyl terminal fusions to FLAG-tagged Aga2. Yeast cells were incubated with either the Erbin-PDZ domain fused to YFP or YFP alone (as a negative control). Greater than 66% of cells are positively labelled using an anti-FLAG antibody, indicating efficient surface display of both PDZ-motifs. 5.33% of cells displaying the Erbin PDZ-binding motif are positively labelled with Erbin-PDZ-YFP versus 0.19% for YFP alone. Negligible binding of either Erbin-PDZ-YFP or YFP alone to cells displaying the Liprin-α1 PDZ-binding motif was observed. These results indicate very specific, though somewhat inefficient binding of the Erbin PDZ domain to a yeast surface-displayed carboxyl terminal PDZ motif. Quantification of binding from three independent experiments analysed by one-way ANOVA and Tukey's multiple comparisons test is shown (right panel). Graph plots the mean % of YFP-labelled cells as individual data points with error bars representing SD. **** $P < 0.0001$ **(B)** Characterization of a Pir2 anchor for carboxyl terminal fusion of proteins of interest. Type 3a' MoClo parts encoding Pir2 with each of three epitope tags were assembled into expression constructs that display the α-GFP Nb as a carboxyl terminal fusion. Surface display and functionality of the nanobody was assessed by flow cytometry using anti-epitope tag anti bodies as well as GFP binding. **(C)** Characterization of a Pir2 anchor in the absence of any POI. Surface display was assessed by flow cytometry as in (B). Schematics of expression constructs indicate the promoter, terminator and signal peptide (SP) sequences used as well as part types (in brackets). The promoter and terminator in (C) are the same as in (A) and (B) but are shown as symbols for brevity.

Figure S2

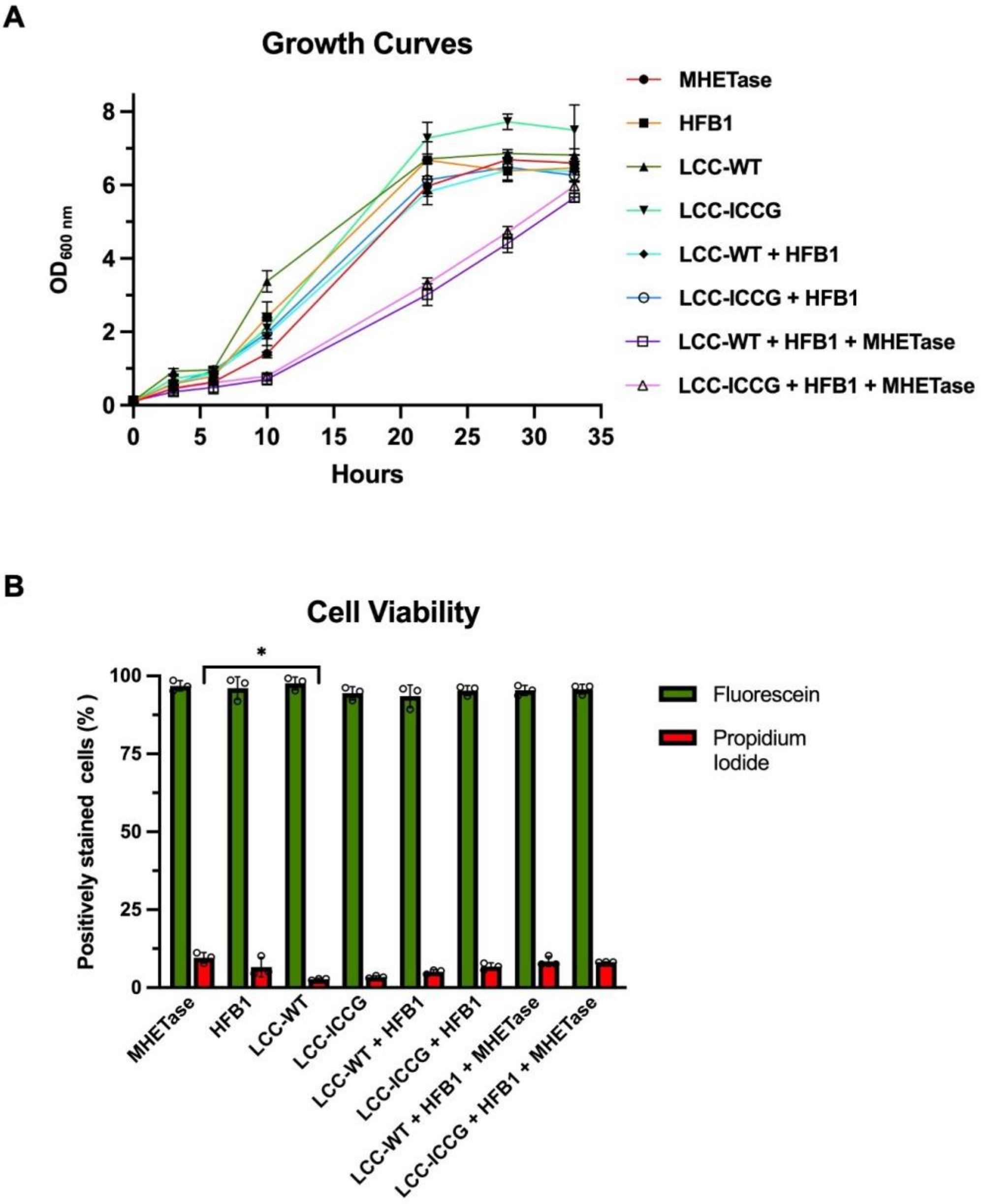

Figure S2 Growth rates and cell viability of single, double and triple MHETase, HFB1 and LCC co-expressing strains

(A) Growth of yeast strains expressing surface display anchored MHETase, HFB1 or the WT/ICCG variants of LCC, either alone, or in the indicated combinations was monitored by measuring optical density of cultures at 600 nm. Strains are the same as those shown in Figure 3. Growth of the triple co-expressing strains was notably slower than the single or double co-expressing ones. Mean values from four biological replicates (with two technical replicates) per condition are shown with error bars representing the standard deviation.

(B) Cell viability was assessed by monitoring the uptake and conversion of fluorescein diacetate to fluorescein as an indicator of metabolically active cells, while dead cells were quantified by propidium iodide staining. Cell staining was measured by flow cytometry. Graphs plot the mean fluorescence measurements from three biological replicates with individual data points and error bars representing SD shown. Differences in cell viability were assessed using Kruskal-Wallis and Dunn's multiple comparisons tests. * indicates $P < 0.05$

Figure S3

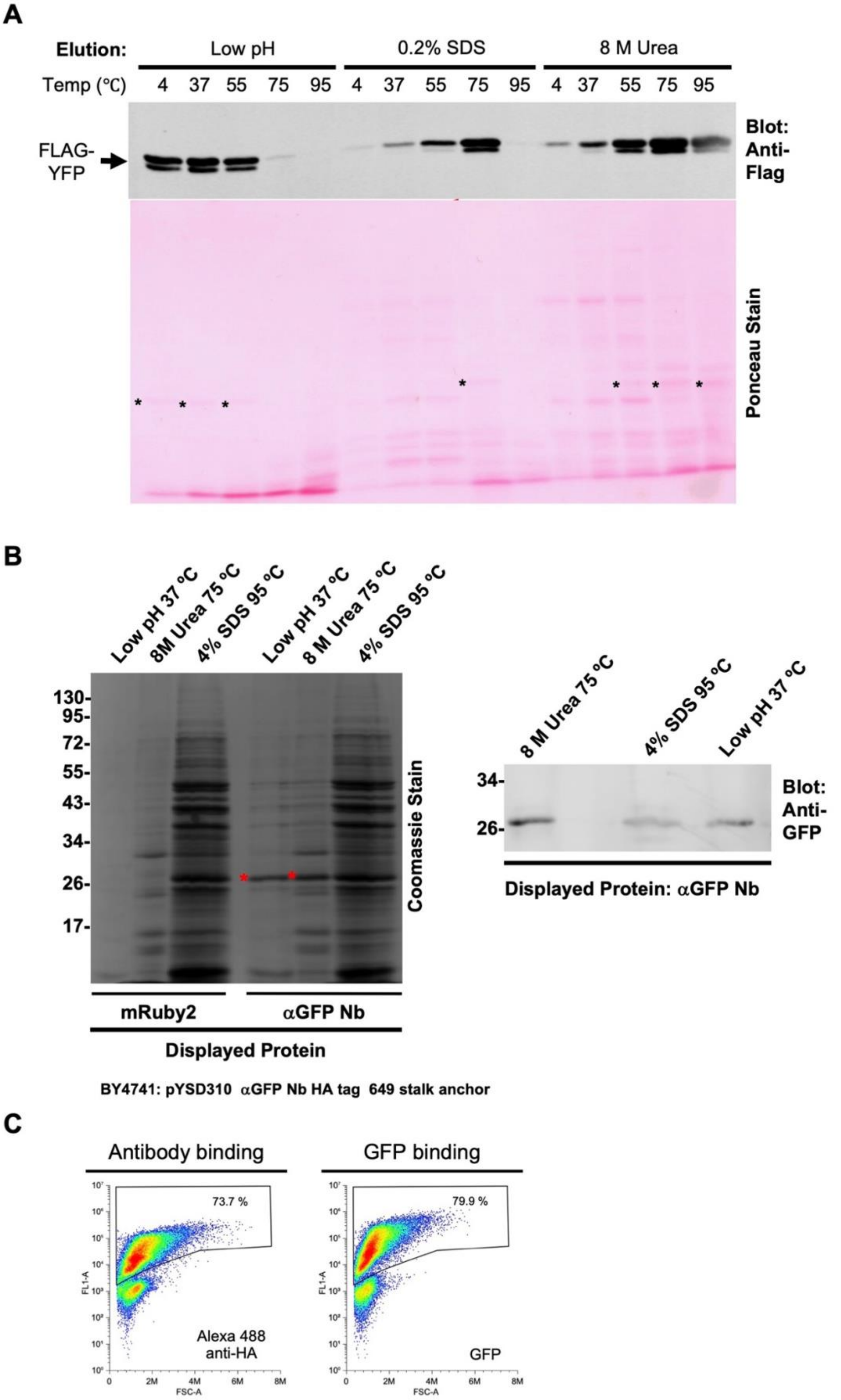
A
Elution:
Low pH
0.2% SDS
8 M Urea
Temp (℃)
4 37 55 75 95 4 37 55 75 95 4 37 55 75 95
FLAG-
YFP
Blot:
Anti-
Flag
Ponceau Stain
B
Low pH 37 °C
8M Urea 75 °C
4% SDS 95 °C
Low pH 37 °C
8 M Urea 75 °C
4% SDS 95 °C
130-
95-
72-
55-
43-
34-
26-
17-
Coomassie Stain
mRuby2
αGFP Nb
Displayed Protein
8 M Urea 75 °C
4% SDS 95 °C
Low pH 37 °C
34-
26-
Blot:
Anti-
GFP
Displayed Protein: αGFP Nb
BY4741: pYSD310 αGFP Nb HA tag 649 stalk anchor
C
Antibody binding
GFP binding
73.7 %
79.9 %
FL1-A
FSC-A
Alexa 488
anti-HA
GFP

Figure S3 Optimization of conditions for immunoprecipitation using yeast displaying an α-GFP Nanobody

(A) Optimization of elution conditions. FLAG-tagged yellow fluorescent protein (YFP) was immunoprecipitated from an *E. coli* cell lysate using 1 x 10^8 yeast cells displaying an α-GFP Nb (α-GFP-Nb yeast). After washing, bound proteins were eluted either by incubating for 5 minutes in either 0.2 M Glycine pH 2.5 (low pH), 0.2% SDS / 100 mM Tris pH 7.5 or 8 M Urea at the indicated temperatures. Eluates were analysed by Ponceau staining and western blotting to detect total protein and FLAG-YFP respectively. **(B)** Specific immunoprecipitation of GFP from a lysate of GFP-expressing *E. coli* cells using 1 x 10^8 yeast cells displaying either an α-GFP Nb or mRuby2 (as a negative control). After washing, bound proteins were eluted either by incubating for 5 minutes in 0.2 M Glycine pH 2.5 at 37 ºC (low pH), 8 M Urea at 75 ºC or SDS-PAGE sample buffer (4% SDS) at 95 ºC. Eluates were analysed by Coomassie staining and western blotting to detect total protein and GFP respectively. GFP is visible by Coomassie staining in the low pH and 8M Urea elutions (red asterisks), while for the 4% SDS/95 ºC elution the GFP band is obscured by the release of large amount of yeast proteins. Western blotting shows elution with 8 M Urea at 75 ºC to yield slightly higher amounts of GFP than the other two elution conditions. **(C)** Surface display and binding activity of α-GFP Nb yeast following medium-term storage at 4 ºC. Flow cytometry analysis was performed on a liquid culture of yeast cells harbouring the pYSD310 plasmid that had been grown overnight in selective media and then stored for two weeks in a fridge.

References

(1) Wiedemann, U., Boisguerin, P., Leben, R., Leitner, D., Krause, G., Moelling, K., Volkmer-Engert, R., and Oschkinat, H. (**2004**) Quantification of PDZ domain specificity, prediction of ligand affinity and rational design of super-binding peptides, *J Mol Biol 343*, 703-718.

(2) Lenihan, J. A., Saha, O., and Young, P. W. (**2017**) Proteomic analysis reveals novel ligands and substrates for LNX1 E3 ubiquitin ligase, *PLoS One 12*, e0187352.
